# Acoustically Altered Speech Perception in Frontotemporal Dementia Syndromes

**DOI:** 10.1212/WNL.0000000000214022

**Published:** 2025-09-11

**Authors:** Jeremy C.S. Johnson, Jessica Jiang, Mai-Carmen Requena-Komuro, Elia Benhamou, Harri Sivasathiaseelan, Annabel Nelson, Doris-Eva Bamiou, Chris J.D. Hardy, Jason D. Warren

**Affiliations:** 1 Dementia Research Centre, Department of Neurodegenerative Disease, UCL Queen Square Institute of Neurology, University College London, United Kingdom;; 2 Basic & Clinical Neurosciences, Kings College London & King's College Hospital NHS Foundation Trust, United Kingdom;; 3 University Medical Center Hamburg-Eppendorf, Hamburg, Germany; and; 4 UCL Ear Institute and UCL/UCLH Biomedical Research Centre, National Institute of Health Research, University College London, United Kingdom.

## Abstract

**Background and Objectives:**

Hearing impairment is a major clinical issue in dementia. Acoustically altered speech (AAS) is a typical real-world listening challenge, but markers of AAS perception, especially in frontotemporal dementias (FTDs), remain undefined. We investigated whether AAS perception differentiates FTD syndromes and predicts real-world hearing symptoms.

**Methods:**

In this cross-sectional case-control study, participants meeting diagnostic criteria for FTD and primary progressive aphasia syndromes were recruited at a UK tertiary center. Participants performed pure-tone audiometry (PTA) and 3 AAS perception tests: speech-in-babble (SIB), spectrally filtered speech (SFS), and time-compressed speech (TCS). Real-world hearing abilities were assessed through the modified Amsterdam Inventory for Auditory Disability and Handicap (mAIAD). Group characteristics were compared using analysis of variance and Fisher exact tests. Regression models adjusted for PTA and Wechsler Abbreviated Scale of Intelligence Matrices scores compared group performance on AAS tests. The Spearman test examined correlations with mAIAD scores.

**Results:**

Among 44 participants, the mean (SD) age was 66 (6.23) years and 13 (29.5%) were female. 29 patients with FTD (6 with nonfluent/agrammatic variant PPA [nfvPPA], 8 with semantic variant PPA [svPPA], and 15 with behavioral variant FTD [bvFTD]) were compared with 15 age-matched controls. Patients with nfvPPA had significantly higher mean PTA thresholds than controls (mean difference 12 decibels [95% CI 0.7–23.4], *p* = 0.034). Those with nfvPPA performed significantly worse on the SFS test than all other groups (vs controls: difference 6.3 [9.4–3.1], *p* < 0.001; vs svPPA group: difference 2.4 [0.5–7.3], *p* = 0.0018; vs bvFTD group: difference 2.0 [1.45–7.0], *p* = 0.0012) and significantly worse on the TCS test than controls and patients with bvFTD (vs controls: difference 88.2 [29.7–146.7], *p* < 0.01; vs bvFTD group: difference 76.1 [26.7–125.5], *p* < 0.01). The svPPA group performed significantly worse than controls on TCS (difference 57.4 [16.38–98.4], *p* = 0.01). Patients with nfvPPA (difference 27.2 [9.3–45.2], *p* < 0.001) and svPPA (difference 25.7 [11.8–39.5], *p* = 0.0013) had significantly worse mAIAD scores than controls. Over the combined patient cohort, mAIAD scores correlated more strongly with AAS than PTA thresholds (TCS, ρ = 0.64, *p* < 0.001; SFS, ρ = 0.53, *p* = 0.003; SIB, ρ = 0.5, *p* = 0.004; PTA, ρ = 0.37, *p* = 0.028).

**Discussion:**

AAS perception stratifies FTD syndromes and constitutes a “real-world audiogram” in these diseases.

## Introduction

Hearing impairment has emerged as a major issue in dementia onset and evolution.^1,2^ However, the nature of the linkage between hearing and dementia and how best to assess this remain unclear.^2-6^ Speech perception is the hearing function most critical for everyday communication, and under the challenging listening conditions of daily life, speech signals are often noisy or otherwise acoustically altered. Perception of such acoustically altered speech (AAS) can be affected early in dementia syndromes, due to both the computational complexity of the neural processing involved and targeting of auditory cortical networks by neurodegenerative pathologies.^2,7-9^ This has important diagnostic and management implications: central deficits of speech perception may signal dementia syndromes and predict everyday communication function but are unlikely to be fully captured by standard audiological tests and interventions directed mainly to peripheral hearing.^2^

The status of AAS perception in different dementia syndromes is not well defined but likely to be particularly relevant to the frontotemporal dementias (FTDs), a complex and heterogeneous group of younger onset neurodegenerative disorders. Communication difficulties with prominent hearing changes are clinical hallmarks of FTD syndromes, most notably in the so-called language-led syndromes of primary progressive aphasia (PPA).^2-4,10^ Patients with nonfluent/agrammatic (nfv) PPA may have marked difficulty processing AAS,^11,12^ because of disrupted neural mechanisms of central auditory pattern perception.^3-5,13^ In semantic variant (sv) PPA, previous work suggests that AAS perception is more dependent on context.^11,12^ Based on clinical experience and neuropsychological evidence,^14^ behavioral variant (bv) FTD is anticipated to produce an auditory phenotype similar to semantic variant PPA (svPPA) but less severe. Measures of AAS perception might, therefore, support the diagnosis and management of impaired communication in FTD and assist in stratifying syndromes within this disease spectrum.

In this study, we assessed the extent to which AAS perception stratifies diagnosis and predicts real-world hearing symptoms in patients presenting with the 3 canonical syndromes of FTD—nfvPPA, svPPA, and bvFTD—compared with healthy, age-matched listeners. We investigated 3 different forms of AAS, each encapsulating a particular kind of everyday listening challenge^7^: speech in background babble, spectrally filtered speech (SFS), and time-compressed speech (TCS). Speech-in-babble [SIB] simulates the well-known “cocktail party effect” whereby a message can be received or a conversation conducted despite an acoustic background of multiple competing talkers. Spectral filtering reduces the acoustic quality of the speech signal principally by removing the fine details carried by higher frequencies, simulating a poor audio connection. Temporally compressing a speech signal captures the difficulty in understanding rapid speech commonly reported by people with PPA. Besides their clinical relevance, we chose these different forms of AAS because the level of acoustic alteration in each can be parameterized to quantify perception accuracy. We further assessed how well PTA and AAS perception measures predicted daily-life hearing difficulties as indexed on a previously validated hearing symptom questionnaire, the modified Amsterdam Inventory for Auditory Disability and Handicap (mAIAD).^15^ Drawing on previous evidence, we hypothesized that measures of AAS perception would differentiate patients with nonfluent/agrammatic variant PPA (nfvPPA) from those with other FTD syndromes and from healthy controls, and that patients with svPPA and behavioral variant FTD (bvFTD) would have qualitatively similar profiles of AAS perception. We further hypothesized that the AAS measures selected here to index aspects of “real-world” hearing would outperform PTA in predicting real-world hearing disability across the patient cohort.

## Methods

### Participant Characteristics

All patients were recruited through a specialist cognitive clinic and fulfilled inclusion criteria for the study, that is, they met consensus clinical diagnostic criteria^16-18^ for the relevant syndrome of mild-to-moderate disease severity (i.e., they were still living in the community and independent in basic activities of daily life). All had a comprehensive neuropsychological assessment (eTable 1) and brain MRI supporting the clinical diagnosis. Age-matched healthy control participants were recruited from the Dementia Research Centre volunteer database. For all participants, exclusion criteria were as follows: non-native speaker of English; significant comorbid cerebrovascular burden on MRI; a history of significant other neurologic, psychiatric, or otological disorders; or inability to repeat or transcribe spoken words from clear speech.

### Hearing Assessment

Details of the test stimuli and procedures used are presented in eAppendix 1.

Pure-tone audiometry (PTA) threshold was calculated as the average minimum threshold in decibels (dB) across all tested frequencies (250, 500, 1,000, 2000, 4,000, and 8,000 Hz).

For assessment of AAS perception, test stimuli were based on 3 different acoustic manipulations of recorded spoken word lists (eFigure 1). For all AAS tests, 50% perception threshold (i.e., the presentation level of the stimulus at which 50% of responses are correct) was determined using an automated “one-up-one-down” staircase paradigm whereby a correct response increased the difficulty level of the stimulus by 1 step and an incorrect response decreased the difficulty by 1 step. The test was terminated once 4 consecutive reversals occurred at a given level. For the SIB test, we adapted a previously described procedure,^19^ varying signal-to-noise ratio (SNR) of target spoken words against unintelligible, multitalker babble: the SNR for 50% correct perception of spoken words was assessed for each participant. For the SFS test, words (eTable 2) were band-pass filtered with a high pass at 350 Hz to remove fundamental frequencies and a low pass at 14 filter levels ranging from 8,000 to 450 Hz: the filter level corresponding to 50% correct perception of spoken words was calculated for each participant. For the TCS test, words were time-compressed digitally at 16 levels ranging from 0% (i.e., no compression) to 300% of original duration separated by steps of 20%: the percentage temporal compression for 50% correct perception of spoken words was calculated for each participant. A non-manipulated reference condition was included in each test. All tests used a uniform response procedure, requiring participants to repeat the spoken word on each trial. Where participants were severely dysarthric, they were able to transcribe rather than repeat the spoken word.

To assess daily-life hearing function, each patient's primary caregiver completed the mAIAD (items listed in eTable 3)^15^; control participants completed the questionnaire themselves.

### Statistical Analysis

Statistical tests were performed using the latest version of R. Group characteristics were compared using analysis of variance (continuous variables) and Fisher exact tests (categorical variables). Multiple linear regression models assessed main effect of the diagnostic group on test scores, covarying for better ear average PTA score and Wechsler Abbreviated Scale of Intelligence (WASI) Matrices score (an index of overall disease severity and executive function); post hoc between-group pairwise comparisons were conducted using the Tukey method to correct for multiple comparisons if the omnibus test was significant. Group-wise correlations between 50% threshold performance level on each hearing test and mAIAD scores were assessed using Spearman rho. An α level of 0.05 was accepted as the statistical significance threshold for all tests.

### Standard Protocol Approvals, Registrations, and Participant Consents

This study was approved by the institutional research ethics committee. All participants gave informed consent in line with Declaration of Helsinki guidelines.

### Data Availability

Anonymized data not published within this article will be made available by request from any qualified investigator.

## Results

Twenty-nine consecutively presenting patients (6 with nfvPPA [mean age 68.5; all male; mean 3 years of symptom duration; mean WASI Matrices score 16.7]; 8 with svPPA [mean age 65.6; 5 male; mean 6.4 years of symptom duration; mean WASI Matrices score 27.4]; 15 with bvFTD [mean age 66.1; 11 male; mean 5.5 years of symptom duration; mean WASI Matrices score 15.7]) and 15 healthy control participants (mean age 65.2; 9 male; mean WASI Matrices score 26.2) met inclusion criteria and participated in the study. No potential participants required exclusion based on the criteria detailed above.

**Figure 1:**
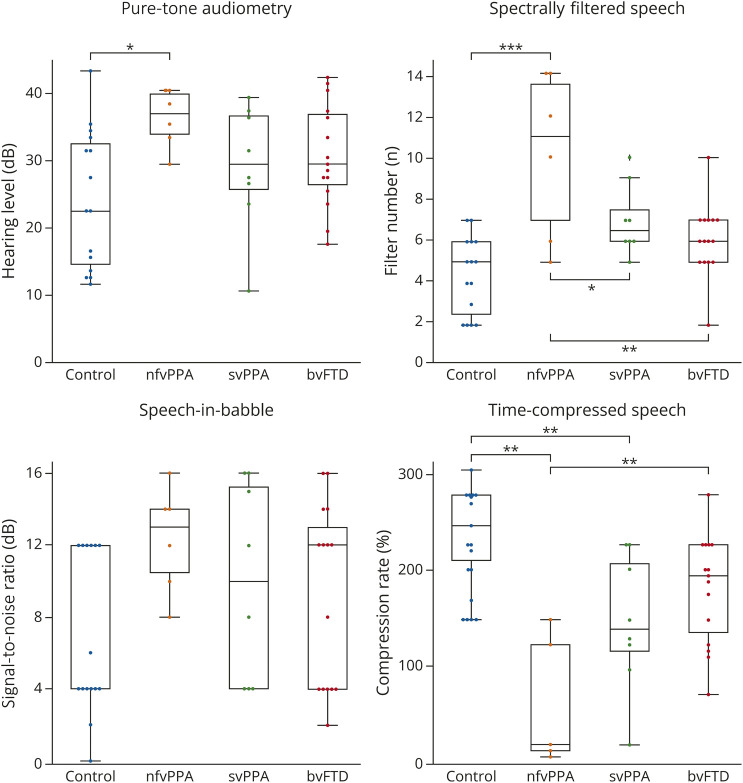
Performance of Participant Groups on Auditory Tests The figure shows box-and-whisker plots of performance profiles on pure-tone audiometry and tests of acoustically altered speech perception in each participant group (text). Pure-tone audiometry scores show the better ear average threshold across frequencies tested (text). Boxes code the interquartile range and whiskers the overall range of scores in each group; the horizontal line in each box represents the median score. Circles represent individual participant scores. Between-group comparisons that reached statistical thresholds are indicated with horizontal square brackets; corresponding *p* values are coded with asterisks as follows: *p < 0.05; **p < 0.01; ***p < 0.001. Performance of patients with nfvPPA on the spectrally filtered and time-compressed speech tests showed least overlap with the healthy control range. Control = healthy control group; svPPA = semantic variant primary progressive aphasia; nfvPPA = nonfluent/agrammatic variant primary progressive aphasia; bvFTD = behavioral variant frontotemporal dementia.

Patient group characteristics and test performance are presented in Table 1 and Figure 1.

**Table 1:** Demographic, Clinical, Cognitive, and Auditory Characteristics of Participant Groups

Characteristic	Controls	nfvPPA group	svPPA group	bvFTD group	Statistic	*p* Value
General						
No	15	6	8	15	NA	NA
Sex (M:F)	9:6	6:0	5:3	11:4	NA	*p* = 0.34
Age (y)	65.2 (6)	68.5 (7.5)	65.6 (7.3)	66.1 (5.7)	F(3,40) 0.40	*p* = 0.76
Handedness (R:L)	13:2	5:1	8:0	14:1	NA	*p* = 0.65
Symptom duration (y)	NA	3.0 (1.6)^a,b^	6.4 (2.0)^a^	5.5 (1.6)^b^	F(2,26) 6.89	*p* < 0.01
Education (y)	15.2 (3.0)	13.2 (2.2)	15.3 (2.1)	12.9 (2.7)	F(3,40) 2.68	*p* = 0.06
MMSE score (/30)	29.6 (0.6)	**22.7 (7.2)**	**22.9 (5.1)**	**23.4 (3.5)**	F(3,37) 8.62	*p* < 0.001
WASI Matrices score (/32)	26.2 (3.0)	**16.7 (11.8)** ^a^	**27.4 (3.2)** ^a,c^	**15.7 (9.0)** ^c^	F(3,38) 8.05	*p* < 0.001
Hearing tests						
PTA (dB)	23.8 (10.4)	**35.8 (4.4)**	28.6 (9.5)	30.3 (7.8)	F(3,40) 3.05	*p* = 0.039
		**12 (0.7–23.4)**	4.3 (−5.5–15.1)	6.5 (−2.10–15.04)		
Speech-in-babble (SNR)^d^	6.9 (4.5)	12.3 (2.9)	12.3 (2.9)	9.2 (5.1)	F(5,36) 2.73	*p* = 0.034
		−3.1 (−10.1–3.9)	−1.9 (−7.3–3.5)	−0.6 (−6.3–5.1)		
Spectrally filtered speech (fn)^e^	4.4 (1.8)	**10.2 (3.9)** ^a,b^	7.0 (1.7)	6.1 (1.7)	F(5,36) 8.14	*p* < 0.001
		**6.3 (3.1–9.4)**	2.4 (−0.1–4.8)	2.03 (−0.5–4.6)		
Time-compressed speech (%)^f^	250 (35.4)	**113 (52.6)** ^b^	**178 (54.8)**	205 (43.5)^b^	F(5,35) 19.3	*p* < 0.0001
		**88.2 (29.77–146.7)**	**57.4 (16.4–98.4)**	12.1 (−31.4–55.7)		
Hearing symptoms						
mAIAD	106.5 (4.7)	**71.7 (14.4)**	**79.5 (15.9)**	86.9 (14.8)	F(5,35) 11.35	*p* < 0.0001
		**27.2 (9.3–45.2)**	**25.7 (11.8–39.5)**	11.4 (−3.43–26.3)		

Abbreviations: bvFTD = behavioral variant frontotemporal dementia; controls = healthy control group; dB = decibels; F = female; fn = frequency channel number; L = left; M = male; mAIAD = modified Amsterdam Inventory for Auditory Disability and Handicap; MMSE = Mini-Mental State Examination; NA = not applicable; nfvPPA = nonfluent/agrammatic variant primary progressive aphasia; PTA = pure-tone audiometry (better ear average threshold for tone detection); R = right; svPPA = semantic variant primary progressive aphasia; WASI = Wechsler Abbreviated Scale of Intelligence; y = years.

No participant had a history of clinically relevant hearing loss or MRI evidence of major comorbid cerebrovascular burden. Group mean (SD) data values are shown unless otherwise indicated; for hearing tests, mean differences for each patient group vs the healthy control group with 95% CIs [in square brackets] are also shown. For MMSE and WASI Matrices, maximum scores are shown after test names (in parentheses). For hearing tests, threshold values are reported (text), and for hearing symptoms, total scores on the mAIAD are given. For each characteristic, omnibus test statistics for overall group effect with *p* values are shown on the right. Significant differences (*p* < 0.05) between groups in post hoc comparisons based on a significant omnibus test are coded as follows: bold, significantly different between patient groups and healthy controls.

a Significantly different between nfvPPA and svPPA groups.

b Significantly different between nfvPPA and bvFTD groups.

c Significantly different between svPPA and bvFTD groups.

d Signal-to-noise ratio (SNR) for 50% correct perception of spoken words against background babble.

e Low-pass filter number (indexed at 14 levels corresponding to bark filters ranging from 8,000 to 450 Hz) for 50% correct perception of spoken words (eAppendix 1).

f Percentage temporal compression of speech signal for 50% correct perception of spoken words.

PTA threshold was significantly increased in the nfvPPA group compared with healthy controls (nfvPPA group, 35.8 (4.4) dB; controls, 23.8 (10.4) dB; mean difference 12 dB ([95% CI 0.7–23.4], *p* = 0.034) but not significantly increased in the other syndromic groups (svPPA group, 28.6 [9.5] dB; mean difference 4.3 [−5.5 to 15.1], *p* = 0.59; bvFTD group, 30.3 [7.8] dB; mean difference 6.5 [−2.10 to 15.04], *p* = 0.20 [Table 1]). Participant groups differed significantly in their performance on the SFS and TCS tests (Table 1). On the SFS test, the nfvPPA group performed significantly worse than the healthy control, svPPA, and bvFTD groups (nfvPPA group, filter number 10.2 [3.9]; controls, 4.4 [1.8], mean difference 6.3 [9.4–3.1], *p* < 0.001; svPPA group, 7.0 [1.7], mean difference 2.4 [0.5–7.3], *p* = 0.0018; bvFTD group, 6.1 [1.7], mean difference 2.0 [1.45–7.0], *p* = 0.0012). On the TCS test, 1 participant with nfvPPA was excluded because they failed to correctly identify any words in the control condition, making their threshold unrecordable. The nfvPPA and svPPA groups performed significantly worse than healthy controls (nfvPPA group % temporal compression 113 [52.6]; controls, 250 [35.4], mean difference 88.2 [29.7–146.7], *p* < 0.01; svPPA group, 178 [54.8], mean difference 57.4 [16.38–98.4], *p* = 0.01), and the nfvPPA group also performed significantly worse than the bvFTD group (205 [43.5], mean difference 76.1 [26.7–125.5], *p* < 0.01). No group-wise comparisons survived correction for multiple comparisons on the SIB test (Table 1).

**Figure 2:**
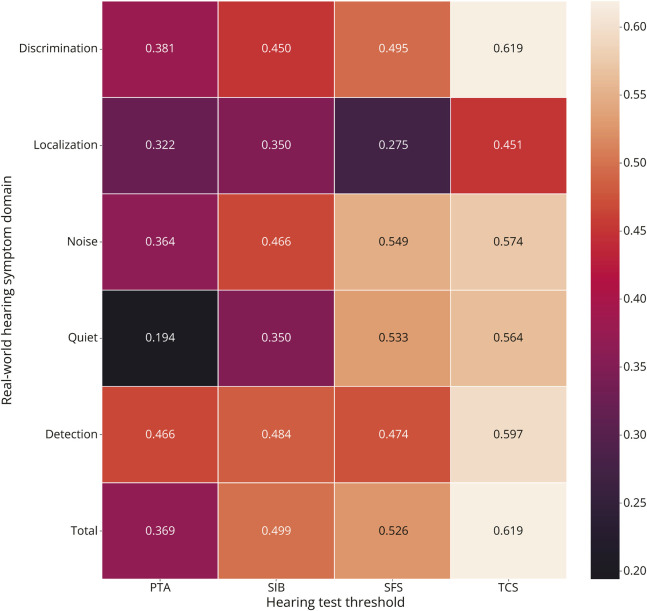
Correlations of Real-World Hearing Symptoms With Auditory Test Performance in the Patient Cohort The figure presents a heatmap of correlations between subdomain scores (y axis) on the modified Amsterdam Inventory for Auditory Disability and Handicap (mAIAD) questionnaire and auditory test thresholds (x axis), across the combined patient cohort. Individual questionnaire items are presented in eTable 3; a heatmap of all correlations is presented in eFigure 2 and corresponding Spearman rho and *p* values are given in eTable 4. mAIAD items are displayed clustered according to the auditory domain they are intended to assess. Each cell shows the Spearman rho correlation coefficient between the score on the relevant questionnaire item and the auditory test score; lighter colors code increasing strength of correlation, as indexed on the color bar (right). Subtotals indicate correlations across all items in that mAIAD auditory domain; total indicates the correlation across all items in the questionnaire. Discrimination = items exploring auditory discrimination (e.g., do you recognize your family members by their voices?); localization = items exploring auditory spatial localisation (e.g., do you hear from what direction a car horn is coming?); noise = items exploring speech understanding in noisy environments (e.g., can you understand a shop assistant in a crowded shop?); quiet = items exploring speech understanding in quiet (e.g., can you understand the presenter of the news on TV?); detection = items exploring sound detection (e.g., can you hear the doorbell at home?); total = correlation coefficient across all items on mAIAD; PTA = pure-tone audiometry (better ear average); SIB = speech-in-babble; SFS = spectrally filtered speech; TCS = time-compressed speech (text and Figure 1).

As indexed by the mAIAD total score (PTA adjusted), the nfvPPA and svPPA groups had significantly worse real-world hearing than healthy controls (controls, 106.5 [4.7]; nfvPPA group, 71.7 [14.4], difference 34.8 [9.3–45.2], *p* < 0.001; svPPA group, 79.5 [159], difference 27.2 [11.8–39.5], *p* = 0.0013; Table 1); there were no significant differences between patient groups. Correlations of mAIAD scores with performance on each of the auditory tests across the combined patent cohort are shown graphically in Figure 2 (full correlation data in eTable 4 and eFigure 2). The mAIAD total score more strongly correlated with 50% threshold performance on the AAS tests than with PTA threshold (TCS, ρ = 0.62, *p* < 0.001; SFS, ρ = 0.53, *p* = 0.003; SIB, ρ = 0.5, *p* = 0.004; PTA, ρ = 0.37, *p* = 0.028). Across individual mAIAD items, the mAIAD score generally more strongly correlated with performance on TCS than on other hearing tests (eTable 4 shows details of statistical analysis).

## Discussion

In this study, we have shown that tests of AAS perception relevant to real-world listening ability can differentiate FTD syndromes from healthy older controls and from one another, after adjusting for peripheral hearing ability and disease severity. The nfvPPA group had the most marked impairment, affecting perception of both spectrally degraded and rapid speech. Furthermore, both nfvPPA and svPPA groups experienced significantly more daily-life hearing difficulties than healthy older controls, corroborating other evidence that these PPA syndromes produce early and salient changes in real-world hearing.^8^ The reduced performance of both PPA groups on the TCS test endorses the bedside observation that patients with PPA struggle to follow rapid talkers—increased load of speech processing mechanisms by TCS exposes language system dysfunction in these syndromes that is relevant to daily-life communication, suggesting a candidate cross-syndromic diagnostic “stress test” for PPA.

The profile of AAS perceptual performance in the nfvPPA group is in line with previous evidence for a clinically relevant, cortical deficit of acoustic signal analysis in this syndrome, affecting the processing of both spectral and temporal features.^3-5,7,13^ These patients had more severe AAS deficits than other syndromic groups and were disadvantaged by reducing spectral detail in the speech signal as well as by increased temporal processing load. This pattern is likely to reflect the involvement of core auditory association cortices as part of peri-Sylvian language network degeneration in nfvPPA^2,3,20,21^: these regions play a fundamental role in representing auditory objects such as phonemes and in trafficking auditory information for further analysis.^22,23^ Patients with nfvPPA further performed worse than healthy controls on PTA. This replicates previous work in the syndrome and is likely to signify a disorder of auditory efferent pathway function rather than a primary cochlear deficit.^5,13^

The deficits observed in the svPPA group are likely to reflect impaired “top-down” predictive decoding of perceptually ambiguous speech signals. We have shown previously that perception of acoustically altered words in svPPA is highly dependent on their predictability.^12^ In this study, the spoken words differed in their frequency (for example, “inkwell” is a lower frequency word than “birthday”; eTable 2) and, therefore, their predictability under acoustic distortion. The primary target of the neurodegenerative process in svPPA is the semantic memory network anchored in the left anterior temporal lobe; however, more posterior left temporal, inferior frontal, and homologous right-sided cortical areas are also implicated and contribute importantly to altered perceptual decoding of speech and other complex acoustic signals in this syndrome compared with the healthy older brain.^24-27^ The profile of AAS test performance in the bvFTD group here was qualitatively similar to that in the svPPA group (albeit somewhat attenuated), in line with the extensive phenotypic overlap between these syndromes.^14,28^ The profile of regional neurodegeneration in bvFTD is heterogeneous but predominantly targets prefrontal and anterior temporal cortices and their subcortical connections,^25,29^ including cingulo-insular pathways involved in decoding degraded speech signals^30^ and hedonic valuation of sounds.^31^ Involvement of auditory cortical areas is typically less extensive and severe in bvFTD than in PPA syndromes, which may explain why AAS perception was less affected in the bvFTD group than in other patient groups in this study.

Our findings further demonstrate that measures of AAS perception in FTD syndromes consistently outperform standard PTA as predictors of daily-life hearing ability in a number of different functional domains, highlighting the limitations of PTA as a measure of real-world hearing in dementia.^32^ It is noteworthy that none of the hearing tests used here was a strong predictor of everyday spatial hearing (Figure 2), emphasizing the functional relevance of our AAS tests for speech perception. This work suggests that bespoke measures of auditory brain function (such as AAS perception) may constitute a “real-world audiogram” for the FTD spectrum, while also illustrating the need to develop additional tests to assess the wider spectrum of real-world hearing functions. From a clinical perspective, the findings underline the importance of asking about hearing symptoms to characterize the full impact of FTD syndromes on patients' daily lives and the need to develop standardized, scalable tests of central hearing (auditory brain) function to guide diagnosis and management. Effective management of hearing impairment in these diseases will entail engaging audiologists and speech and language therapists for communication strategies and acoustic environmental modifications, as well as development of “smart” assistive listening devices that enhance speech perception in complex listening environments.^2,7^

This study builds on a growing body of evidence for impaired AAS perception and central hearing deficits more broadly in major dementias.^2,3,11,21,26,33,34^ Limitations of this study include the relatively small cohort size, cross-sectional design, and lack of direct neuroanatomical or histopathologic correlation. AAS measures warrant validation in larger and more diverse patient cohorts, incorporating neuroanatomical and neuropathologic analyses. It is noteworthy that all syndromic groups in this study showed wide individual variation in AAS perceptual performance (Figure 1); the factors that drive this variability are not known, but understanding them will be critical if auditory measures are to be useful as biomarkers. Longitudinal studies will be particularly important, to evaluate the feasibility and utility of real-world hearing changes as early markers of FTD and other dementia syndromes.^2^

## Glossary

AAS: acoustically altered speech
bvFTD: behavioral variant FTD
DB: decibel
FTD: frontotemporal dementia
mAIAD: modified Amsterdam Inventory for Auditory Disability and Handicap
nfvPPA: nonfluent/agrammatic variant PPA
PPA: primary progressive aphasia
PTA: pure-tone audiometry
SFS: spectrally filtered speech
SIB: speech-in-babble
SNR: signal-to-noise ratio
svPPA: semantic variant PPA
TCS: time-compressed speech
